# The Association of Death Receptors and TGF-β1 Expression in Urothelial Bladder Cancer and Their Prognostic Significance

**DOI:** 10.3390/biomedicines12051123

**Published:** 2024-05-18

**Authors:** Slavica Stojnev, Irena Conic, Ana Ristic Petrovic, Ivan Petkovic, Milica Radic, Miljan Krstic, Ljubinka Jankovic Velickovic

**Affiliations:** 1 Center for Pathology, University Clinical Center Nis, 18000 Nis, Serbia; 2 Department of Pathology, Faculty of Medicine, University of Nis, 18000 Nis, Serbia; 3 Clinic of Oncology, University Clinical Center Nis, 18000 Nis, Serbia; irenaconic@yahoo.com (I.C.);; 4 Department of Oncology, Faculty of Medicine, University of Nis, 18000 Nis, Serbia

**Keywords:** death receptor, apoptosis, urothelial bladder cancer, FAS, TGF-β1, prognosis

## Abstract

Death receptor signalization that triggers the extrinsic apoptotic pathway and TGF-β1 have important roles in urothelial carcinogenesis, with a complex interplay between them. The aim of this research was to assess the association of death receptors DR4, DR5, and FAS as well as TGF-β1 immunohistochemical expression with the clinicopathological characteristics of urothelial bladder cancer (UBC) and to evaluate their prognostic significance. The decrease or loss of death receptors’ expression was significantly associated with muscle-invasive tumors, while non-invasive UBC often retains the expression of death receptors, which are mutually strongly linked. High DR4 expression is a marker of low-grade tumors and UBC associated with exposition to known carcinogens. Conversely, TGF-β1 was significantly associated with high tumor grade and advanced stage. High expression of DR4 and FAS indicates longer overall survival. High TGF-β1 signifies an inferior outcome and is an independent predictor of adverse prognosis in UBC patients. This study reveals the expression profile of death receptors in UBC and their possible interconnection with TGF-β1 and indicates independent prognostic significance of high FAS and TGF-β1 expression in UBC, which may contribute to deciphering the enigma of UBC heterogeneity in light of the rapid development of novel and effective therapeutic approaches, including targeting of the TRAIL-induced apoptotic pathway.

## 1. Introduction

Urothelial bladder cancer (UBC) is one of the most significant genitourinary cancers with a high impact on the healthcare system. It is the fourth most common malignancy in men and is associated with high morbidity and mortality [1,2]. UBC comprises a wide spectrum of neoplasms, from non-invasive tumors prone to recurrence that commit a patient to life-long cystoscopic surveillance, to aggressive tumors with high metastatic rates. Recent advances in UBC research and novel insights into molecular characterization and biological heterogeneity of this cancer have warranted an urgent need for further discovery and validation of diagnostic, prognostic, and predictive biomarkers.

Deregulation of apoptosis is a fundamental process in carcinogenesis [3,4]. Death receptors are members of the tumor necrosis factor receptor superfamily that contain a conserved death domain and signal apoptosis, following the binding of appropriate ligands [4,5,6,7]. The reports about complex roles of death receptor pathways in the regulation of cancer invasion and metastasis are somewhat contradictory [7,8]. Most extensively studied and undoubtedly powerful in executing deadly signals are death receptors DR4 (TRAILR1), DR5 (TRAILR2), and FAS (CD95) [7,9,10].

Death receptors DR4 and DR5 are activated by the binding of TRAIL (TNF-related apoptosis-inducing ligand), while FAS-mediated apoptosis is triggered by FAS ligand (FASL). Tumor cells are much more sensitive to TRAIL-triggered apoptosis than normal cells and the molecular basis of this selective sensitivity has not yet been elucidated [8,11,12,13]. In conditions of preserved immunity, FAS-mediated apoptosis plays an important role in the elimination of tumor cells. In tumor progression, the level of soluble FASL increases, which inhibits FAS-mediated tumoricidal activity of cytotoxic T lymphocytes and NK cells and decreases FAS receptor expression levels. Loss of FAS reduces the sensitivity of tumor cells to T-cell cytotoxicity and promotes the ability of tumor cells to evade immune surveillance and facilitates the process of tumor progression and metastasis [3,14]. In bladder cancer, a downregulation of FAS was noted in cancer cells compared to the surrounding normal urothelium [15,16]. Loss of FAS expression was found to significantly correlate with worse prognosis in patients with urothelial carcinomas of the bladder, ureter, and renal pelvis [16]. Apparently, proper identification and clarification of the exact apoptotic machinery and pathways in different tumor cell types may ameliorate the design of more efficacious targeted cancer treatments [1,3,17].

Pleiotropic roles of TGF-β in carcinogenesis have been widely studied. The tumor suppressor and cytostatic effects exerted by TGF-β1 on target cells include inhibition of the cell cycle, prevention of cell immortalization, and the induction of apoptosis. TGF-β1 is the key activator of apoptosis in many normal and tumor cells [18,19]. However, the molecular mechanisms and signaling pathways underlying the pro-apoptotic effects of TGF-β have not yet been precisely characterized. Studies on animal models suggested that TGF-β1 induces apoptosis through the TRAIL/death receptor system [20,21]. During tumor progression, increased secretion of TGF-β in tumor cells, as well as paracrine secretion from surrounding stromal cells, stimulates tumor invasiveness and metastasis followed by immunosuppression, which is mediated by the FASL/FAS system. TGF-β and FASL signaling contribute together to ensure immune privilege in the tumor microenvironment [21]. Moreover, it was found that TGF-β1 can activate FAS receptors in a FASL-independent manner and induce apoptosis in cancer cells [22].

Death receptor signalization and TGF-β have important roles in cancer invasion and metastasis and there is a complex interplay and multiple interconnections between them. The significance of death receptors in UBC has not been fully elucidated so far, neither has their relationship with TGF-β1, a potent multifaceted actor in carcinogenesis. The aim of this research was to assess the association of death receptors DR4, DR5, and FAS and TGF-β1 expression with the clinicopathological characteristics of UBC, their mutual correlation, and to evaluate their prognostic significance.

## 2. Materials and Methods

### 2.1. Patients and Histopathologic Analysis

The present study comprised tissue samples obtained from 647 patients with bladder cancer who underwent transurethral resection of bladder tumor (TURBT) during a 6-year period in the Clinic of Urology, University Clinical Center Nis, Serbia. Pathohistological diagnosis of all tumors was made at the Center for Pathology, University Clinical Center Nis, according to the WHO classification (WHO, 2022, 5th edition [23]) and TNM pathological staging system (TNM classification 2016, 8th edition [24]). A detailed medical record was available for all patients included in the study. The median follow-up of the patients was 61 months (25 to 130 months). Follow-up time was expressed as the number of months, from the day of diagnostic TURBT to the last checkup doctor visit or death outcome. Cancer-specific death was designated if the fatal outcome occurred because of locally advanced or disseminated bladder cancer and it did not include mortality caused by other neoplastic or non-neoplastic causes.

The average patients’ age was 66.4 ± 9.9 years, with the predominance of male patients who consisted 77.1% of the study population. The majority of the patients (62.1%) were active or former cigarette smokers, with at least ten pack-years of smoking history. Occupational exposition to known carcinogens involved in urothelial carcinogenesis, including aromatic amines, polycyclic aromatic hydrocarbons, and heavy metals, was present in 7.4% of the patients. At the time of diagnosis, there were 497 patients with non-muscle-invasive urothelial bladder cancer (NMIBC), out of which 201 were pTa and 296 pT1 superficially invasive carcinomas, while 150 patients had muscle-invasive disease. None of the cases of non-invasive carcinomas were classified as carcinoma in situ because carcinoma in situ was constantly diagnosed in the surrounding of infiltrative urothelial carcinoma, not as a solely neoplastic lesion in the resected material. Furthermore, all tumors were classified as low-grade and high-grade tumors. The presence of divergent differentiation and variant morphology of the tumor was noted. More than 10% of the UBC was classified in this group. Moreover, the presence of prominent cystitis in adjacent mucosa, as well as the abundance of mononuclear or polymorphonuclear inflammatory infiltrate in tumor stroma, was also noted.

### 2.2. Immunohistochemical Analysis

Following the detailed pathohistological diagnosis and precise recording of every parameter of interest, tissue microarray blocks (TMA) were constructed using the manual tissue arrayer (Arraymold Paraffin Tissue Microarrayer, Arraymold, UT, USA) for the purpose of immunohistochemical analysis. Two core samples with a diameter of 2 mm were selected from each case and incorporated in the recipient TMA blocks, together with the adequate control samples of normal bladder mucosa, which were obtained from surgically resected bladder specimens from the patients who underwent pelvic surgery for causes other than UBC. Three μm thick TMA sections were deparaffinized in xylene and rehydrated in a graded ethanol series. A standard avidin–biotin immunoperoxidase complex detection system was used and liquid 3,3′-diaminobenzidine (DAB) was applied as a chromogen, allowing the positive staining reaction in the form of brown precipitate in the respective cellular compartment (membrane/cytoplasm). Every staining procedure included appropriate negative and positive controls.

Immunohistochemical analysis was performed using the primary antibodies to DR4 (rabbit polyclonal antibody to DR4 at dilution of 1:100, ab8414, Abcam, Cambridge, UK), DR5 (rabbit polyclonal antibody to DR5 at dilution of 1:200, ab8416, Abcam, Cambridge, UK), FAS (mouse monoclonal antibody to FAS at dilution of 1:100, Santa Cruz Biotechnology, Santa Cruz, CA, USA), and TGF-β1 (mouse monoclonal antibody at dilution of 1:40, clone TGFB17, Leica Biosystems, Newcastle, UK). The analysis and interpretation of immunohistochemical stains were performed on the light microscope (Olympus BX43, Olympus Corporation, Tokyo, Japan) and digital photographs that were acquired using the imaging system (Olympus cellSens platform standard, Olympus Corporation, Tokyo, Japan). For each core tumor sample, the percentage of stained tumor cells was calculated. Staining intensity was graded using a scale of 0 to 3 (0, no staining; 1, weak; 2, moderate; and 3, intense). For the purposes of statistical analysis, the immunoexpression status of the examined markers was dichotomized into high expression and low expression, which included absent staining or expression under the designated threshold. Death receptor expression (DR4, DR5, and FAS) was considered high if at least ≥25% of tumor cells showed positive expression of moderate or strong brown color intensity. The immunoexpression of TGF-β1 was recorded as high if at least 50% of cancer cells were stained with intermediate or strong intensity.

### 2.3. Statistical Analyses

The expression of immunohistochemical markers and clinicopathologic features were evaluated for association by the χ^2^ test with Yates’s correction and two-tailed distribution and the Fisher exact test. For each investigated marker, the Kaplan–Meier survival curve was constructed to compare the patients with tumors of high or low expression. The differences between survival curves were tested for statistical significance by a log-rank test. Cox regression analysis with the enter method was applied to assess the relationship between the survival of the patients and explanatory variables. All data analyses were performed using the Statistical Package for Social Sciences, version 20.0 statistical software (SPSS, Chicago, IL, USA). A *p*-value of 0.05 or less was considered indicative of a statistically significant difference.

## 3. Results

### 3.1. Expression of Death Receptors DR4, DR5, and FAS in Relation to Clinicopathologic Characteristics

**Figure 1:**
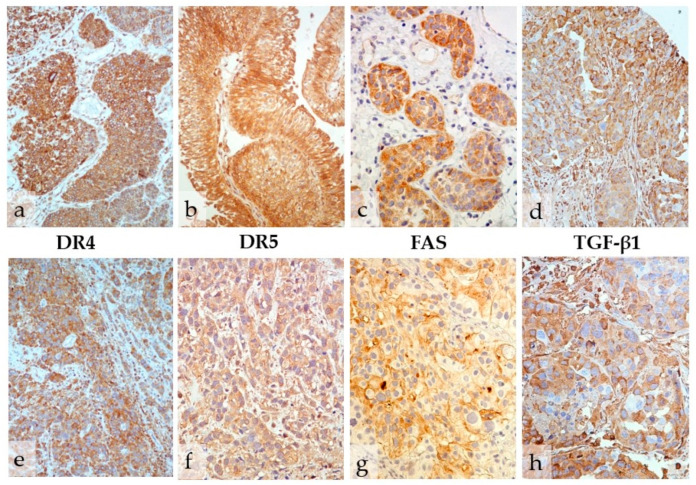
Immunohistochemical expression of death receptors DR4, DR5, and FAS and TGF-β1 in infiltrative pT1 (**a**–**d**) and muscle-invasive pT2 urothelial bladder cancer (**e**–**h**). High immunohistochemical expression of markers is shown (400×).

The high expression of death receptors DR4, DR5, and FAS was observed in 70.2%, 69.9%, and 54.7% of tumors, respectively (Table 1). DR4 staining was mostly cytoplasmic and finely granular, followed by membranous and mixed cytoplasmic/membranous staining patterns. The DR5 expression profile was quite similar; however, the intensity of the reaction was generally weaker than DR4. FAS displayed predominantly moderate expression in the form of membranous and cytoplasmic patterns, often with a distinct golden hue of the staining (Figure 1; Supplement S1, Figure S1). Immunohistochemical staining of the control non-neoplastic urothelium is shown in the Supplementary Material (Supplement S1, Figure S2).

High DR4 expression significantly correlated with tumors affecting patients with professional exposition to known carcinogens (Table 1). High DR4 was observed in 83.3% of carcinogen-associated UBC opposite to 69.1% of sporadic tumors. Conversely, high DR4 was more frequent in tumors of non-smoker patients (73.5% vs. 68.2%); however, this difference was not statistically significant.

DR4 was the only death receptor significantly associated with low tumor grade (*p* < 0.001), where 71% of tumors with loss of DR4 were high-grade carcinomas (Table 2). Low or absent expression of DR4, DR5, and FAS was significantly associated with advanced tumor stage and muscle-invasive disease (*p* < 0.001, *p* = 0.33, and *p* = 0.25, respectively).

Loss of DR4 showed a linear correlation with the increasing stage: DR4 had low expression in 16.9% of pTa, 32.1% pT1, and 42.7% of pT2 tumors. FAS expression also decreased with tumor progression: low FAS was observed in 40.3% pTa, 44.6% pT1, and 53.3% pT2 cancers. In the case of DR5, the largest proportion of decreased expression was among muscle-invasive tumors (37.3%), followed by non-invasive neoplasms (32.3%), while tumors that invaded lamina propria had the lowest rate of DR5 loss (25%). High FAS expression was more frequently found in invasive UBC associated with carcinoma in situ (*p* = 0.038), as well as in tumors with lymphocyte-rich stroma (*p* = 0.033). Urothelial carcinoma with divergent differentiation and variant histology more frequently showed a decrease in DR4 than tumors with classic features (37.9% vs. 28.6%); however, this difference was not statistically significant (*p* = 0.051).

At the designated endpoint of the follow-up period, 285 patients (44.0%) had died and the cause of death was declared as cancer-specific in 30.9% of the patients. Loss of DR4 and FAS indicated death outcomes in cancer patients (Table 3). DR4 and FAS showed inverse correlation to cancer-specific death (*p* = 0.002, and *p* = 0.001, respectively). In terms of treatment, DR4 high tumors were more frequently treated solely with TURBT with or without intravesical instillation of mitomycin. The loss of all death receptor expression is a strong predictor of radical cystectomy in the patients’ follow-up management.

### 3.2. Expression of TGF-β1 in Relation to Clinicopathologic Characteristics

High expression of TGF-β1 was found in 66.5% of the tumors, predominantly in older patients and non-smokers (*p* = 0.01, and *p* = 0.014, respectively) (Table 1). TGF-β1 overexpression was significantly associated with high tumor grade and advanced pathologic stage (*p* < 0.001, respectively). Although a significant portion of low-grade non-invasive papillary urothelial carcinoma demonstrated uniform intense staining, the majority of TGF-β1 high tumors were high-grade neoplasms (68.1%). Out of all the high-grade tumors, high TGF-β1 expression could be observed in 75.7%. The proportion of tumor cells expressing TGF-β1 rose with the advancing stage: high TGF-β1 was found in 47.8% of non-invasive tumors, 73.6% of early invasive, and 77.3% of muscle-invasive carcinoma (Table 2).

Overexpression of TGF-β1 directly and strongly correlated to cancer-specific death (*p* = 0.001). There was no significant association with tumor recurrence. In addition, patients with TGF-β1 positive tumors had a higher probability of being treated with radical surgical interventions or chemoradiotherapy (*p* = 0.008, respectively) and a lower chance of receiving only immunotherapy with Calmette-Guerin bacillus (*p* = 0.016) (Table 3).

### 3.3. Association of Death Receptors DR4, DR5, and FAS, and TGF-β1 Expression

**Figure 2:**
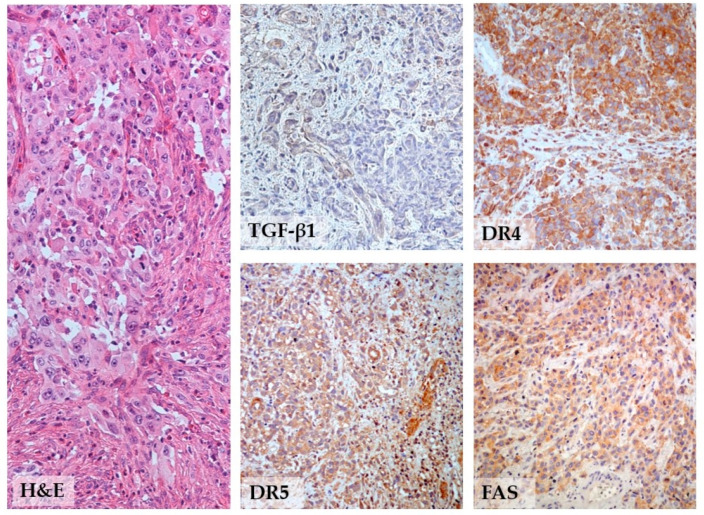
Representative example of the immunoprofile of invasive urothelial bladder cancer with infiltration of lamina propria. This high-grade pT1 UBC had low TGF-β1 expression and simultaneous high diffuse expression of the three death receptors, DR4, DR5, and FAS (200×).

The analysis of markers’ expression correlation (Table 4) indicated a strong relationship between DR4 and DR5 (*p* < 0.001), with more than half of the tumors showing high expression of both death receptors (54.4%) (Figure 2). Among tumors (14.4%) with loss of both receptors, muscle-invasive tumors were predominant (49.5%). TGF-β1 was linked only to DR5 (*p* < 0.006), while DR4 high and DR4 low tumors showed quite equalized expression of TGF-β1. FAS was significantly associated with both DR4 and DR5 (*p* < 0.001, and *p* = 0.003, respectively), as well as with TGF-β1 (*p* = 0.03). Triple-negative tumors with loss of all three death receptors (57/647) included muscle-invasive tumors (42.1%) and superficially invasive pT1 tumors (57.9%).

### 3.4. Association of Death Receptors DR4, DR5, and FAS and TGF-β1 and Overall Survival

**Figure 3:**
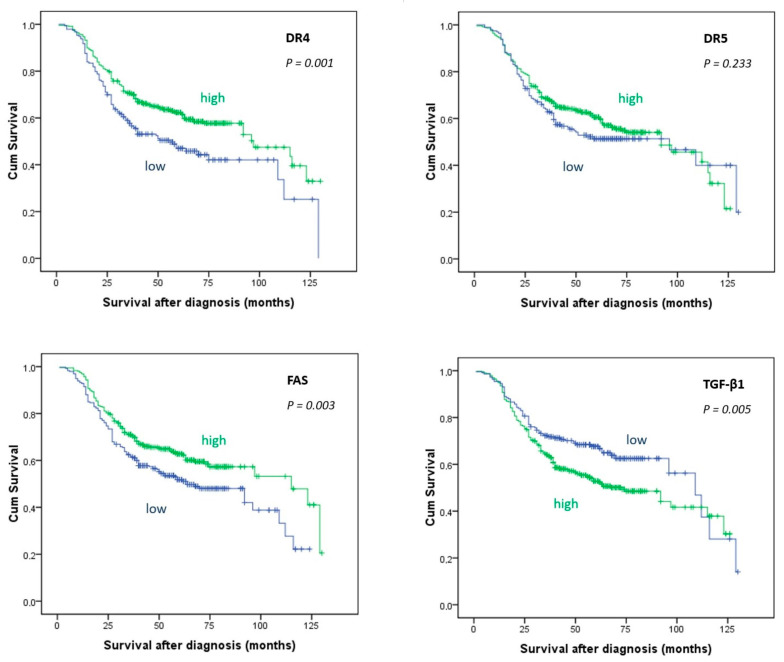
Kaplan–Meier survival curves showing overall survival in 647 patients with urothelial bladder cancer in relation to expression of TGF-β and death receptors DR4, DR5, and FAS in cancer cells. Log Rank (Mantel-Cox) test.

Survival analyses showed that high expression of death receptors DR4 and FAS was significantly linked to better prognosis of the UBC patients (*p* = 0.001 and *p* = 0.003, respectively). Inversely, high TGF-β1 expression was significantly associated with worse overall survival (*p* = 0.005) (Figure 3). Expression of DR5 did not show a significant association with patients’ overall survival (*p* = 0.233). Immunohistochemical expression of TGF-β1, as well as high death receptors (DR4, DR5, and FAS), did not show a significant correlation with recurrence-free survival (*p* = 0.866, *p* = 0.535, *p* = 0.149, and *p* = 0.585, respectively).

Cox regression analysis of a significant model that included categorical independent variables demonstrated in Table 5 identified FAS and TGF-β1 expression as the independent predictors of patients’ survival. Namely, besides the tumor grade and pathologic stage, after the adjustment for other explanatory variables in the model, the two markers retained the prognostic significance. The hazard ratio suggested that the risk of death outcome decreased by 75% (95% CI: 59.3% to 94.8%) in patients with UBC, demonstrating high FAS expression.

## 4. Discussion

This research included 647 urothelial carcinomas of the urinary bladder consecutively diagnosed during a six-year period in patients from Serbia. Almost half of patients with NMIBC (49.1%) developed disease recurrence during the follow-up period, out of which a third of the patients even had multiple recurrences. The proportion of muscle-invasive tumors at initial diagnostic TURBT was 23.2%, which is similar to results in other European populations [25,26]. The unpredictable nature of this frequent cancer warrants committed research of every important aspect of the tumor.

It is well known that many cancers arise after exposure to various exogenous carcinogens with different routes of entrance into the circulation, namely inhalation, ingestion, or direct contact [27,28]. Tobacco smoke is responsible for at least 50% of UBC. Occupational exposure to known carcinogens is the second most frequent cause of exogenously driven urothelial carcinogenesis. Genetic variations in detoxifying enzymes underlie the susceptibility to bladder cancer in people professionally exposed to components in dye, rubber, oil, and other industries. High DR4 expression significantly correlated with tumors in patients with professional exposition to known carcinogens. High DR4 was observed in 83.3% of carcinogen-associated UBC opposite to 69.1% of sporadic tumors. Retention of death receptor expression may suggest the redundancy of extrinsic apoptotic pathways in occupational exposure-associated bladder carcinogenesis. An interesting inverse correlation was found between smoking status and TGF-β1 expression. The majority of non-smokers (71.8%) had TGF-β1 high tumors, which may suggest diverse TGF-β1 pathways in carcinogenesis.

The TRAIL apoptosis induction system has attracted the attention of researchers since its discovery because of its ability to trigger apoptosis primarily in tumor cells [8,17,20]. Although TRAIL can interact with several different receptors, only DR4 and DR5 contain the intracellular death domain necessary for apoptotic signal transmission after ligand binding to the receptor. Recent studies showed that the expression level of death receptors in UBC cells correlates with sensitivity to TRAIL-induced apoptosis [29,30]. Sensitivity to TRAIL in vitro diminishes with decreasing expression of death receptors DR4 and DR5 on the cell surface. The studies interrogating DR4 and DR5 in human bladder cancer material are very scarce [30,31]. Analysis of immunohistochemical expression of DR4 and DR5 on paraffin samples of 229 bladder tumors obtained by TURBT showed that these markers were expressed in 75.1% and 74.2% of urothelial carcinomas, respectively [31]. These results are very similar to our findings, where the expression was only discreetly lower.

In this study, DR4 expression strongly correlated with low histological tumor grade, while DR4 and DR5 both showed a strong association with pathological stage. Consistent with the results of previous studies [30,31], reduction in or loss of death receptor expression for TRAIL correlates significantly with muscle-invasive disease. In addition, DR4 expression has been identified as a negative marker of cancer-specific mortality. Patients with high DR4 expression have a significantly longer overall survival, as much as 14 months longer on average, than patients with DR4-low tumors. However, multivariate analysis did not confirm the significance of DR4 as an independent prognostic factor for survival in bladder cancer. Although in a previously published study [31] the expression of DR4 and DR5 was associated with a longer recurrence-free survival time, our results did not confirm such an association.

Because of the property of selective and powerful activation of programmed cell death, the therapeutic potential of TRAIL and death receptors are the subject of intense research. Recent proteogenomic study revealed that *FGFR3*-mutated UBC are sensitive to TRAIL-induced apoptosis, which certainly warrants future clinical studies [32]. Mechanisms of sensitivity and resistance to TRAIL-induced apoptosis are complex and can only be partly explained by the expression level of death receptors on the surface of tumor cells. Downregulation of death receptors has been described in many cancers, including colorectal and triple-negative breast cancer [13,33]. Bladder urothelial carcinoma cell lines sensitive to TRAIL-induced apoptosis were found to have increased death receptor expression, while TRAIL-resistant cancer lines had decreased DR5 receptor expression [30]. Cytotoxic agents that can increase the expression of death receptors could sensitize cancer cells to TRAIL-induced apoptosis [13].

Bladder cancer is associated with mutations of the FAS death receptor in almost 30% of cases, most often missense mutations in the region encoding the death domain responsible for the transduction of apoptotic signals. *FAS/FASL* gene polymorphisms have recently been found to have prognostic significance in assessing the therapeutic response in BCG-treated patients with high-risk NMIBC [34]. BCG therapy is not effective in 30–40% of treated patients and the identification of non-responders is of great importance because the delay of more aggressive therapy, e.g., early cystectomy, is avoided. Tumor cells can become less sensitive to FAS-induced apoptosis by down-regulation of FAS receptors and thereby promote immune privilege that enables tumor growth and progression. On the other hand, pro-inflammatory cytokines and, above all, TGF-β, can induce upregulation of FASL expression and enable tumor cells to evade the host immune response [14]. Cytokine production in the specific tumor microenvironment, dominated by hypoxia and acidosis, supports tumor cell survival via FAS downregulation in tumor cells. Resistance to apoptosis due to the FAS blockade may play a significant role in tumorigenesis and tumor progression [16,35].

An immunohistochemical study of FAS and FASL, which included 40 UBC of different stages, indicated a significant reduction in the expression of FAS and increased expression of FASL in bladder cancer tissue. FAS was positive in only 37.5% of cancers, while FAS was expressed in 87.5% of surrounding peritumoral urothelium and in normal bladder tissue [15]. Our results were in agreement with previously publisher results in urothelial cancers [16]. It was found that FAS expression decreases with progressive tumor stage. The association of low FAS and worse prognosis recognized in this study could be related to the evasion of apoptosis by shutting down a potent FAS-induced mechanism. However, the FAS expression level does not necessarily predict cancer cell susceptibility to apoptosis [16].

The effects of TGF-β1 on apoptosis depends on the type of cells and on the context in which the effect is realized and is based on the activity and involvement of different intracellular signaling pathways in a certain cell in its specific microenvironment [19,36]. The main mechanism by which TGF-β recruits TRAIL and activates the extrinsic pathway of apoptosis is the transcriptional activation of the *TRAIL* gene because the *TRAIL* promoter is a direct and primary target of TGF-β signaling [20,21]. On the other hand, TGF-β does not contribute to the stabilization of the nascent mRNA transcript [20,21]. In vitro, studies indicated that TGF-β significantly induces the transcription of *TRAIL* and increases its expression on the surface of tumor cells but that the treatment of tumor cells in culture with TGF-β does not lead to an increase in either the mRNA level or the surface protein expression of DR4 and DR5 receptors [20]. The results of our research support the fact that TGF-β does not induce the death receptors, although correlation analysis of the immunohistochemical expression levels of TGF-β1 and DR5 showed significant association. Notwithstanding, in the survival analysis, DR4 had a protective impact on patients’ survival, opposite to the negative effect of TGF-β1.

Although it is known that TGF-β increases motility and stimulates the accumulation and recruitment of immune cells in the tumor stroma [37,38,39], while directly inhibiting their anti-tumor effector functions, the results of this study did not indicate a significant correlation of extensive inflammatory infiltrate in the tumor stroma with TGF-β1 expression in tumor cells. The absence of a statistically significant correlation does not exclude the influence of TGF-β on the structure and function of immunocompetent cells in the tumor. Only high FAS correlated to the abundant stromal lymphocytic response. In physiological conditions, FASL is expressed on cytotoxic T lymphocytes and natural killer cells, while in cancer it can be expressed in tumor cells. The FASL/FAS receptor system engages in killing tumor-infiltrating lymphocytes, i.e., tumor counterattack against the host. It was found that the loss of FAS expression inversely correlates with the rate of apoptosis in tumor-infiltrating lymphocytes. Decreased immunohistochemical expression of FAS in urothelial tumors is significantly associated with higher histological grade and higher pathological tumor stage [15,16], while our results only confirmed the association with muscle-invasive disease. Among the molecules involved in FAS signaling, the death receptor FAS, i.e., its deficiency, has been shown to be the most important in the progression of bladder tumors [16], which is in accordance with our finding that high FAS predicts superior outcome and longer overall survival in UBC.

One of the main goals of this study was to determine the prognostic significance of TGF-β1 immunohistochemical expression. The results showed that TGF-β1 expression is an independent predictor of inferior overall survival and worse prognosis. It was found that there is a significant difference in the survival of patients with TGF-β1 negative tumors or tumors with low expression, who had an average of 8 months longer survival than patients with TGF-β1-high tumors. High expression of TGF-β1 is significantly associated with cancer-specific mortality, in agreement with previously published data [40].

To our knowledge, this is the largest study of prognostic value of death receptors DR4, DR5, and FAS, and TGF-β1 in UBC.

Limitations that need to be considered derive from the inherent frailty of methodology based on immunohistochemistry. Three experienced pathologists independently reviewed the slides to make the optimal assessment of each tumor staining.

## 5. Conclusions

This study indicated that the expression of death receptors DR4, DR5, and FAS in urothelial bladder cancer is significantly decreased in muscle-invasive UBC. Non-invasive pTa tumors often retain the expression of death receptors, which are mutually strongly associated. High DR4 expression is more frequently found in low-grade tumors, as well as in UBC associated with exposition to known carcinogens, while high FAS indicates carcinoma in situ adjacent to the invasive tumor. DR4 and FAS are negative and TGF-β1 is a positive predictor of cancer-specific death. High expression of DR4 and FAS indicates longer overall survival. High TGF-β1 signifies an inferior outcome and is an independent predictor of adverse prognosis in UBC patients. The present study that evaluated the expression profile of death receptors in UBC, their possible association with TGF-β and prognostic impact, may be a small but valuable contribution in deciphering the enigma of UBC heterogeneity in the light of rapid development of novel and effective therapeutic approaches, including the targeting of TRAIL-induced apoptotic pathway.

## Figures and Tables

**Table 1:** Association of death receptors DR4, DR5, and FAS and TGF-β1 expression with demographic and epidemiologic features of the patients with urothelial bladder cancer.

		DR4		DR5		FAS		TGF-β1	
Expression		(High)	*p*	(High)	*p*	(High)	*p*	(High)	*p*
total N (%)	647 (100)	454 (70.2)		452 (69.9)		354 (54.7)		430 (66.5)	
Gender									
Female	148 (22.9)	109 (24.0)	0.171	106 (23.5)	0.336	77 (21.8)	0.256	106 (24.7)	0.078
Male	499 (77.1)	345 (76.0)		346 (76.5)		277 (78.2)		324 (75.3)	
Age									
<66	279 (43.1)	195 (43.0)	0.480	195 (43.1)	0.529	146 (41.2)	0.163	171 (39.8)	**0.010**
≥66	368 (56.9)	259 (57.0)		257 (56.9)		208 (58.8)		259 (60.2)	
Cigarette Smoking									
Yes	402 (62.1)	274 (60.4)	0.089	275 (60.8)	0.173	212 (59.8)	0.112	254 (59.1)	**0.014**
No	245 (37.9)	180 (39.6)		177 (39.2)		142 (40.2)		176 (40.9)	
Exposition to known carcinogens									
Yes	48 (7.4)	40 (8.9)	**0.024**	33 (7.3)	0.488	30 (8.5)	0.165	41 (6.7)	0.221
No	599 (92.6)	414 (91.2)		419 (92.7)		324 (91.5)		401 (93.3)	

A value of *p* ≤ 0.05 was considered statistically significant (in bold).

**Table 2:** Association of death receptors DR4, DR5, and FAS, and TGF-β1 expression with pathologic features of bladder cancer.

		DR4		DR5		FAS		TGF-β1	
Expression		(High)	*p*	(High)	*p*	(high)	*p*	(High)	*p*
total N (%)	647 (100)	454 (70.2)		452 (69.9)		354 (54.7)		430 (66.5)	
Tumor grade									
Low	260 (40.2)	204 (44.9)	**<0.001**	189 (41.8)	0.115	173 (48.9)	0.388	137 (31.9)	**<0.001**
High	387 (59.8)	250 (55.1)		263 (58.2)		213 (60.1)		293 (68.1)	
Pathologic stage									
pTa	201 (31.1)	167 (36.8)	**<0.001**	136 (30.1)	0.467	120 (33.9)	0.089	96 (22.3)	**<0.001**
pT1	296 (45.7)	201 (44.3)	0.483	222 (49.1)	0.005	164 (46.3)	0.752	218 (45.7)	**<0.001**
≥pT2	150 (23.2)	86 (18.9)	**<0.001**	94 (20.8)	0.033	70 (19.8)	**0.025**	116 (27.0)	**<0.001**
Carcinoma in situ									
Yes	47 (7.3)	30 (6.6)	0.204	31 (6.9)	0.324	32 (9.1)	**0.038**	36 (8.4)	0.083
No	600 (92.7)	424 (93.4)		421 (93.1)		322 (90.9)		394 (91.6)	
Divergent differentiation/Variant tumor morphology									
Negative	560 (86.6)	400 (88.1)	0.051	389 (86.1)	0.337	310 (87.6)	0.236	369 (85.8)	0.258
Positive	87 (13.4)	54 (11.9)		63 (13.9)		44 (12.4)		61 (14.2)	
Cystitis									
Yes	106 (16.4)	69 (15.2)	0.129	69 (15.3)	0.146	62 (17.5)	0.228	63 (14.7)	0.060
No	541 (83.6)	385 (84.8)		383 (84.7)		292 (82.5)		367 (85.3)	
Extensive lymphocytic inflammatory infiltrate									
Present	45 (7.0)	31 (6.8)	0.482	29 (6.4)	0.254	31 (8.8)	**0.033**	35 (8.1)	0.063
Absent	602 (93.0)	423 (93.2)		423 (93.6)		323 (91.2)		395 (91.9)	
Extensive polymorphonuclear inflammatory infiltrate									
Present	13 (2.0)	11 (2.4)	0.203	12 (2.7)	0.061	4 (1.1)	0.071	10 (2.3)	0.315
Absent	634 (98.0)	443 (97.6)		440 (97.3)		350 (98.9)		420 (97.7)	

A value of *p* ≤ 0.05 was considered statistically significant (in bold).

**Table 3:** Association of death receptors DR4, DR5, and FAS, and TGF-β1 expression with clinicopathologic features of bladder cancer.

		DR4		DR5		FAS		TGF-β1	
Expression		(High)	*p*	(High)	*p*	(High)	*p*	(High)	*p*
total N (%)	647 (100)	454 (70.2)		452 (69.9)		354 (54.7)		430 (66.5)	
Clinical presentation									
Hematuria	543 (83.9)	348 (84.6)	0.279	386 (85.4)	0.077	295 (83.3)	0.366	360 (83.7)	0.469
Other	104 (16.1)	70 (15.4)		66 (14.6)		59 (16.7)		70 (16.3)	
Recurrence									
Yes	244 (37.7)	172 (37.9)	0.481	178 (39.4)	0.106	141 (39.8)	0.127	160 (37.2)	0.387
No	403 (62.3)	282 (62.1)		274 (60.6)		213 (60.2)		270 (62.8)	
Cancer specific death									
Yes	200 (30.9)	124 (27.3)	**0.002**	135 (29.9)	0.216	90 (25.4)	**0.001**	153 (35.6)	**0.001**
Other	85 (13.1)	58 (12.8)		56 (12.4)		48 (13.6)		54 (12.6)	
Alive	362 (56.0)	272 (59.9)		261 (57.7)		216 (61.0)		223 (51.8)	
Treatment									
TURBT *	122 (18.9)	95 (20.9)	**0.024**	80 (17.7)	0.150	74 (20.9)	0.086	72 (16.7)	**0.035**
Intravesical BCG	303 (46.8)	218 (48.0)	0.200	221 (48.9)	0.065	175 (49.4)	0.084	188 (43.7)	**0.016**
Cystectomy	101 (15.6)	53 (11.7)	**<0.001**	63 (13.9)	**0.049**	44 (12.4)	**0.010**	78 (18.2)	**0.008**
Chemo/radioth	121 (18.7)	88 (19.4)	0.286	88 (19.5)	0.259	61 (17.2)	0.170	92 (21.4)	**0.008**

A value of *p* ≤ 0.05 was considered statistically significant (in bold). * transurethral resection with or without intravesical instillation of a single dose of mitomycin.

**Table 4:** Association of death receptors and TGF-β1 in urothelial bladder cancer.

		TGF-β1		DR4		DR5		FAS	
Expression		High	*p*	High	*p*	High	*p*	High	*p*
Total (n (%))	647 (100)	430 (66.5)		454 (70.2)		452 (69.9)		354 (54.7)	
TGF-β									
High	430 (66.5)	-		298 (65.6)	0.279	315 (69.7)	**0.006**	247 (69.8)	**0.030**
Low	217 (33.5)	-		156 (34.4)		137 (30.3)		107 (30.2)	
DR4									
High	454 (70.2)	298 (69.3)	0.279	-		352 (77.9)	**<0.001**	276 (77.9)	**<0.001**
Low	193 (29.8)	132 (30.7)		-		100 (22.1)		78 (22.1)	
DR5									
High	452 (69.9)	315 (73.3)	**0.006**	352 (77.5)	**<0.001**	-		264 (74.6)	**0.003**
Low	195 (30.1)	115 (26.7)		102 (22.5)		-		90 (25.4)	
FAS									
High	354 (54.7)	247 (57.4)	**0.030**	276 (60.8)	**<0.001**	264 (58.4)	**0.003**	-	
Low	293 (45.3)	183 (42.6)		178 (39.2)		188 (41.6)		-	

A value of *p* ≤ 0.05 was considered statistically significant (in bold).

**Table 5:** Multivariate analysis of the prognostic factors by Cox regression analysis showing significant variables with independent influence on the overall survival for 647 patients with urothelial bladder cancer (Cox regression model of UBC-specific survival).

		Overall Survival			
Parameter	B	HR	95%CI		*p*-Value
			Lower	Upper	
Age (≥66)	0.713	2.039	1.546	2.690	**<0.001**
Tumor grade (high)	0.701	2.016	1.375	2.956	**<0.001**
Pathologic stage T1	0.728	2.071	1.032	3.247	**0.002**
Pathologic stage ≥T2	1.560	4.758	2.906	7.789	**<0.001**
TGF-β1 (high)	0.801	2.228	1.567	3.167	**<0.001**
DR4 (high)	−0.087	0. 916	0.707	1.189	0.511
DR5 (high)	−0.060	0. 942	0.726	1.223	0.654
Fas (high)	−0.299	0.741	0.581	0.946	**0.016**

A value of *p* ≤ 0.05 was considered statistically significant (in bold).

## Data Availability

Data are contained within the article.

## Supplementary Materials

The following supporting information can be downloaded at: https://www.mdpi.com/article/10.3390/biomedicines12051123/s1, Figure S1. The examples of immunohistochemical staining of death receptors in urothelial bladder cancer. In the upper row are representative photomicrographs of high and low DR4 expression in non-muscle-invasive bladder cancer (NMIBC). The lower row contains photomicrographs of the same case of muscle-invasive bladder cancer (MIBC) with low DR4 and low DR5 expression. Figure S2. Immunohistochemical staining of death receptors and TGF-β1 in non-neoplastic bladder mucosa. In non-neoplastic urothelium, death receptors DR4 and DR5 showed diffuse granular cytoplasmic and membranous expression, while FAS displayed fine, delicate membranous staining with accentuation in umbrella cells. TGF-β1 stained membranes of basal and/or apical layers, but never full thickness of the urothelium. In addition, DR4 stained stromal immune cells, while TGF-β1 showed reactivity in stromal mesenchymal and some inflammatory cells.
