# Supplementary material for: The Association of Death Receptors and TGF-β1 Expression in Urothelial Bladder Cancer and Their Prognostic Significance
Source: Biomedicines. 2024 May 18;12(5):1123. doi: 10.3390/biomedicines12051123 (PMC11117556; doi:10.3390/biomedicines12051123)
Supplement: Supplementary file 1 [file biomedicines-12-01123-s001.zip › biomedicines-3003019-supplementary.pdf]

Supplement S1

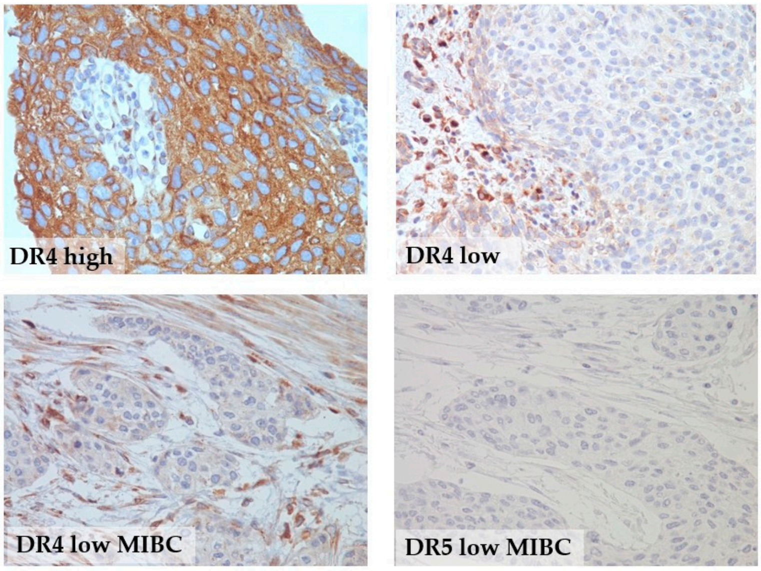

Figure S1**.** The examples of immunohistochemical staining of death receptors in urothelial bladder cancer. In the upper row are representative photomicrographs of high and low DR4 expression in non-muscle-invasive bladder cancer (NMIBC). The lower row contains photomicrographs of the same case of muscle-invasive bladder cancer (MIBC) with low DR4 and low DR5 expression.

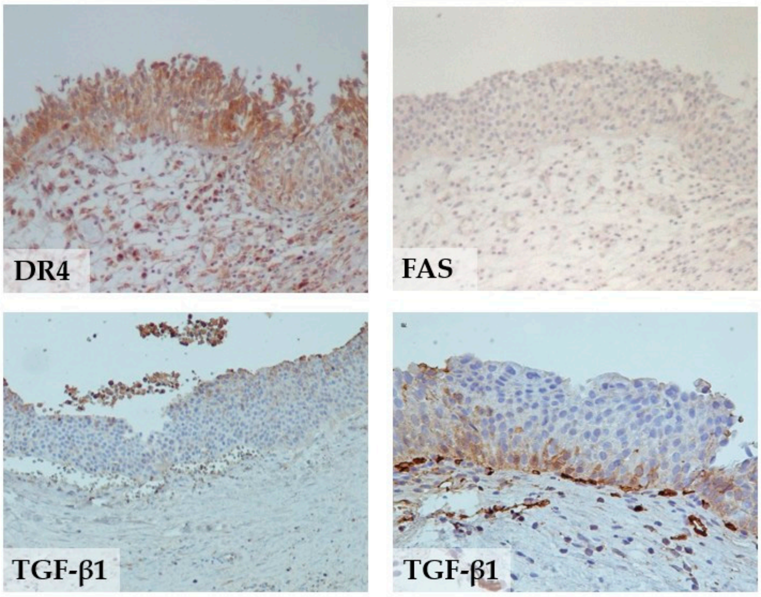

Figure S2. Immunohistochemical staining of death receptors and TGF-β1 in non-neoplastic bladder mucosa. In non-neoplastic urothelium, death receptors DR4 and DR5 showed diffuse granular cytoplasmic and membranous expression, while FAS displayed fine, delicate membranous staining with accentuation in umbrella cells. TGF-β1 stained membranes of basal and/or apical layers, but never full thickness of the urothelium. In addition, DR4 stained stromal immune cells, while TGF-β1 showed reactivity in stromal mesenchymal and some inflammatory cells.
